# Hypertension and Aging Affect Liver Sulfur Metabolism in Rats

**DOI:** 10.3390/cells10051238

**Published:** 2021-05-18

**Authors:** Dominika Szlęzak, Patrycja Bronowicka-Adamska, Tomasz Hutsch, Marcin Ufnal, Maria Wróbel

**Affiliations:** 1 Jagiellonian University Medical College, Faculty of Medicine, Chair of Medical Biochemistry, 7 Kopernika St., 31-034 Kraków, Poland; dominika.szlezak@uj.edu.pl (D.S.); patrycja.bronowicka-adamska@uj.edu.pl (P.B.-A.); 2 Laboratory of the Centre for Preclinical Research, Department of Physiology and Experimental Pathophysiology, Medical University of Warsaw, 1B Banacha St., 02-097 Warsaw, Poland; t.hutsch@yahoo.com (T.H.); mufnal@wum.edu.pl (M.U.); 3 Veterinary Diagnostic Laboratory ALAB Bioscience, ALAB Plus Sp. z o.o., 13 Krucza St., 05-090 Rybie, Poland

**Keywords:** liver, hypertension, aging, hydrogen sulfide, cystathionine gamma-lyase, 3-mercaptopyruvate sulfurtransferase, thiosulfate sulfurtransferase (rhodanese)

## Abstract

Hypertension and age are key risk factors for cardiovascular morbidity and mortality. Hydrogen sulfide (H_2_S), a gaseous transmitter, contributes significantly to regulating arterial blood pressure and aging processes. This study evaluated the effects of hypertension and aging on the hepatic metabolism of sulfur-containing compounds, the activity of the enzymes involved in sulfur homeostasis, and the liver’s ability to generate H_2_S. Livers isolated from 16- and 60-week-old normotensive Wistar Kyoto rats (WKY) and Spontaneously Hypertensive Rats (SHR) were used to evaluate gene expression using RT-PCR, and the activity of enzymes participating in H_2_S metabolism, including thiosulfate sulfurtransferase (rhodanese; TST), cystathionine gamma-lyase (CTH), and 3-mercaptopyruvate sulfurtransferase (MPST). The levels of cysteine, cystine, reduced and oxidized glutathione were measured using RP-HPLC. SHR livers from both age groups showed a higher capacity to generate H_2_S than livers from WKY. The gene expression and activity of enzymes involved in sulfur metabolism differed between WKY and SHR, and between the age groups. For example, 16-week-old SHR had significantly higher activity of TST than 16-week-old WKY. Furthermore, differences between younger and older WKY rats in the expression and/or activity of TST and MPST were present. In conclusion, our study shows that arterial hypertension and aging affect hepatic sulfur metabolism and H_2_S production in rats. These findings pave the way for interventional studies evaluating a potential causal relation between liver sulfur metabolism, hypertension and aging.

## 1. Introduction

Chronic diseases (including cardiovascular diseases) contribute to 60% of the total number of worldwide deaths [1]. One of the main causes of cardiovascular disorders is hypertension, which can contribute to diseases of many organs of the body, including heart, aorta, blood vessels, and kidneys. The risk of developing cardiovascular disorders increases with age [2]. The molecular aspects of aging include genomic instability, telomere attrition, cellular senescence, and mitochondrial dysfunction. These changes result in impaired body functions, and consequently lead to death [3].

**Figure 1:**
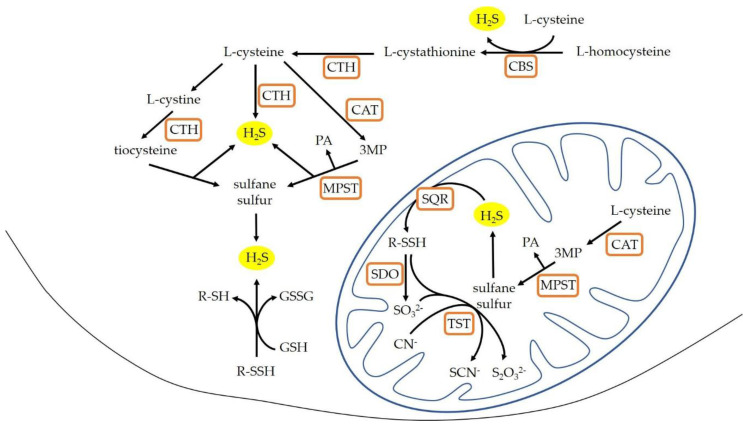
H_2_S production in the mammalian cells (modified according to [5,7]). CAT: cysteine aminotransferase; CBS: cystathionine β-lyase; CTH: cystathionine gamma-lyase; GSH: glutathione reduced form; GSSG: glutathione oxidized form; MPST: 3-mercaptopyruvate sulfurtransferase; 3MP: 3-mercaptopyruvate; PA: pyruvate; R-SH: thiols; R-SSH: persulfides; SQR: sulfide quinone oxidoreductase; SDO: sulfur dioxygenase; TST: rhodanese.

Hydrogen sulfide (H_2_S) has a significant effect on the cardiovascular system function and participates in blood pressure regulation [4]. H_2_S is endogenously synthesized from l-cysteine in a reaction catalyzed by cytosol enzymes: cystathionine beta-synthase (CBS, EC 4.2.1.22) and cystathionine gamma-lyase (CTH, EC 4.4.1.1) with the participation of the cofactor—pyridoxal phosphate (vitamin B_6_). Another enzyme that is involved in the synthesis of hydrogen sulfide is 3-mercaptopyruvate sulfurtransferase (MPST, EC 2.8.1.2). MPST catalyzes the desulfuration of 3-mercaptopyruvate (3-MP) resulting from the conversion of L-cysteine by cysteine aminotransferase (CAT, EC 2.6.1.3). Compared to CBS and CTH, MPST is present both in the cytosol, and in the mitochondria. As a result of reactions catalyzed by these enzymes, compounds containing sulfane sulfur are formed. H_2_S is produced by the reduction of sulfane sulfur, or reduction of thiosulfates and persulfides, which are formed in the presence of the mitochondrial enzyme thiosulfate sulfurtransferase (rhodanese; TST, EC 2.8.1.1). Additionally, H_2_S can be produced by nonenzymatic reduction of persulfides in the presence of glutathione [5,6] (Figure 1).

The liver is a major organ responsible for maintaining homeostasis of the entire organism. The most important functions of the liver include synthesis of plasma proteins and clotting factors, regulation of carbohydrates and fats metabolism, neutralization of toxins and drugs metabolism [8,9]. Additionally, the synthesis of glutathione occurs in the liver being its major producer and exporter [10]. In physiological conditions the liver is the most important organ that produces H_2_S [11]. Endogenous H_2_S participates in regulation of glucose and lipid metabolism, hepatic mitochondrial bioenergetics, and regulation of oxidative stress [11,12]. Additionally, H_2_S can play a protective role in hepatic diseases such as fibrosis and cirrhosis [13], hepatic ischemia-reperfusion injury [14], and liver cancer [15].

Liver morphology and metabolism change throughout life. With age, the liver volume and the blood flow decreases, volume of hepatocytes changes, area of smooth endoplasmic reticulum decreases, and a lower number and dysfunction of mitochondria are observed [16]. Aging promotes accumulation of lipids in different tissues, also in liver, and is associated with the accumulation of reactive oxygen species (ROS) as well [9,17].

In this study, we hypothesized that normotensive and hypertensive rats differ in the hepatic metabolism of sulfur-containing compounds, activity of enzymes involved in the production of hydrogen sulfide, and the liver ability to generate H_2_S. Additionally, we examined the influence of age on these parameters.

## 2. Materials and Methods

### 2.1. Animals

This was a post hoc study evaluating sulfur metabolism in livers harvested from control rats in the study by Huc and others [18], approved by the I Local Bioethical Committee in Warsaw (permission: 100/2016). The experiment involved male normotensive Wistar Kyoto (WKY) rats and male Spontaneously Hypertensive Rats (SHRs) of different ages (16- or 60-week-old) from Central Laboratory of Experimental Animals, Medical University of Warsaw. Rats were housed in groups of two to three animals in polypropylene cages with environmental enrichment on a 12:12 light:dark schedule, at 22 °C and 55% humidity, and received a standard diet and water ad libitum. Once they reached the suitable age, rats were anesthetized with intraperitoneal injection of urethane (1.5 g/kg body weight). Hemodynamic parameters including arterial blood pressure were measured via an arterial catheter using Biopac MP 150 (BiopacSystems, Goleta, CA, USA) [18]. After the measurements, blood from the right ventricle of the heart was taken, and rats were killed by decapitation. Tissues collected during dissection were washed out in cold saline, and immediately frozen in liquid nitrogen, and stored at −80 °C for further procedures. Animal euthanasia and tissue collection were carried out in the operating room of the Department of Physiology and Experimental Pathophysiology at the Medical University in Warsaw.

### 2.2. Experimental Groups

For this study animals of different ages were divided into four groups: 16-week-old Wistar Kyoto rats (16-WKY)—7 animals; 60-week-old Wistar Kyoto rats (60-WKY)—7 animals; 16-week-old Spontaneously Hypertensive Rats (16-SHR)—8 animals; 60-week-old Spontaneously Hypertensive Rats (60-SHR)—8 animals.

### 2.3. Histopathological Examination

The harvested liver fragments were fixed in a buffered solution of 10% formalin. The preserved organ fragments were macroscopically examined and then dehydrated in graded ethanol and xylene baths. The dehydrated sections (measuring 3–4 µm) were then embedded in paraffin wax. The sections were stained with hematoxylin and eosin (H-E). Microscopic evaluation was performed at 10× and 40× magnification and the sample was photographed. The structure of the liver and abomasum wall tissue were examined using an Axiolab A5 light microscope with Axiocam 208 color and ZEN 3.0 software (Zeiss, Jena, Germany).

### 2.4. Tissue Homogenates

To determine the enzymes activity (CTH, MPST, TST, CBS), the level of sulfane sulfur, and protein content, the tissue samples were weighed and homogenized in ice-cold 0.1 M phosphate buffer pH 7.5 (1 g tissue/4 mL solution) for 1 min at 8000–9500 rpm using a blender homogenizer. The homogenates were centrifuged at 1600× *g* for 10 min.

For the RP-HPLC (reversed-phase high-performance liquid chromatography) method, the tissues were weighed and homogenized at 8000–9500 rpm in ice-cold 10% PCA/1 mM BPDS (1 g tissue/3 mL solution). The homogenates were then centrifuged for 10 min at 4 °C, 1400× *g*. The supernatants were either immediately used for assays or stored at −80 °C until RP-HPLC analysis.

### 2.5. Expression of CTH, MPST, TST, and CBS Gene

#### 2.5.1. Isolation of RNA

Total RNA was extracted from tissues using the Tri Reagent (Lab Empire, Rzeszów, Poland), according to the protocol provided by the manufacturer. Extracted RNA was suspended in nuclease-free water. The quality of the RNA samples was determined by spectrophotometric analysis as the ratio of the absorbance: 260 nm/280 nm and electrophoresis in 2.5% agarose gel with ethidium bromide. The RNA solutions were stored at −80 °C until further procedures.

#### 2.5.2. Reverse Transcription of RNA

Total RNA from the tissue samples was reverse-transcribed using a First-Stand complementary DNA (cDNA) synthesis kit according to the manufacturer instructions (Promega, Madison, WI, USA). For the reverse transcription (RT), 3 μg of the total RNA was mixed with 1 μL of Oligo(dT)_18_ primer (0.5 μg/µL) and nuclease-free water and heated in a 70 °C heat block for five minutes. After preincubation, the reverse transcription reaction mix containing 4 μL GoScript^TM^ 5× reaction buffer, 3 μL 25 mM MgCl_2_, 1 μL deoxyribonucleotide triphosphates (dNTPs, 10 mM), 1 μL Recombinant RNases Ribonuclease Inhibitor (20 U/μL) and 1 μL GoScript^TM^ Reverse Transcriptase (160 U/μL), was added to the mixture with RNA in the total volume of 20 µL. The mixture was first incubated for 5 min at 25 °C, then for 60 min at 42 °C, and for the final 15 min at 70 °C. The solution of cDNA was stored at −20 °C.

#### 2.5.3. Polymerase Chain Reaction (PCR)

Amplification of cDNA samples was run in mixture containing 2 µL of synthesized cDNA, 10 µM of gene-specific primer pair, 5 U/µL DNA polymerase in 20 mM buffer Tris-HCl pH 8.0 (includes 20 mM MgCl_2_), 10 mM each of dNTPs, and nuclease-free water in total volume 25 µL.

For the CTH gene, after an initial denaturation at 94 °C (5 min), amplification was performed under the following conditions: 95 °C (30 s), 56 °C (30 s), 72 °C (2 min) for 36 cycles, with a final extension at 72 °C (8 min). The primer sequences were as follows: forward 5′-TTTGTATACAGCCGCTCTGGA-3′, and reverse 5ʹ-ACAAGCTTGGTCTGTGGTGT-3′ (product: 290 bp). For the MPST and GAPDH (Glyceraldehyde-3-phosphate dehydrogenase) gene, PCR cycling conditions were 94 °C (5 min) for one cycle, 95 °C (30 s), 54 °C (30 s), and 72°C (1 min) for 28 cycles, with a final extension at 72 °C (8 min). The primer sequences were as follows: MPST forward 5′-TCCTGGGTGGAGTGGTACAT-3′, and MPST reverse 5′-GTGAAACAAGCTAGGTGGGC-3′ (339 bp) [19] and GAPDH forward 5′-CCGCATCTTCTTGTGCAGTG-3′, and GAPDH reverse 5′-ACCAGCTTCCCATTCTCAGC-3′ (239 bp) [20]. For the TST gene, after an initial denaturation at 94 °C (5 min), amplification was performed under the following conditions: 94 °C (30 s), 55.6 °C (30 s), and 72 °C (2 min) for 38 cycles, with a final extension at 72 °C (8 min). The primer sequences were as follows: forward 5′-CTCTATCGAGCGCTGGTCTC-3′ and reverse 5′-TCGTAAGGCGAAGTCGTGTC-3′ (200 bp). For the CBS gene, after an initial denaturation at 95 °C (10 min), amplification was performed under the following conditions: 94 °C (20 s), 60 °C (1 min), and 72 °C (1 min) for 40 cycles, with a final extension at 72 °C (5 min) [19]. The primer sequences were as follows: forward 5′-CTGTGAAGGGCTATCGCTGC-3′ and reverse 5′-CTGGCATTGCGGTACTGGTC-3′ (205 bp) [21].

GAPDH gene was used as an internal control to normalize all the samples for potential variations in mRNA content. The PCR reaction products were separated electrophoretically in 2.5% agarose gel, visualized with ethidium bromide under UV light, and photographed. The band intensity was examined by densitometric analysis using the system of documentation and computer analysis UVI-KS 4000i/ImagePC (Syngen Biotech, Wrocław, Poland).

### 2.6. Enzyme Assay

The CTH activity was determined according to Matsuo and Greenberg method [22] with modification by Czubak and others [23]. The incubation mixture contained 25 µL 1.3 mM PLP, 25 µL 13 mM EDTA, 250 µL 45 mM cystathionine solution in 0.1 M phosphate buffer (pH 7.5)—2.5 mg cystathionine per sample, 75 µL tissue homogenate, and 0.1 M phosphate buffer (pH 7.5) containing 0.05 mM 2-mercaptoethanol, in a final volume of 650 µL. The reaction was stopped after 30 min of incubation at 37 °C by placing 125 µL incubation mixture in 25 µL 1.2 M PCA. The samples were centrifuged at 1600× *g* for 10 min, and 25 µL of supernatant was transferred to 625 µL 0.2 mM NADH solution, and stored at 37 °C for measurement. The control samples were prepared in the same way as the examined samples, but without 45 mM cystathionine. After 10 s of the measurement (A_340 nm_), 25 µL (9.06 IU) l-lactic dehydrogenase were added, and the measurement was continued until 180 s. The difference between the initial value of absorbance (before LDH addition) and the lowest value (after LDH addition) corresponded to the amount of α-ketobutyrate formed in the course of the γ-cystathionase reaction. The enzyme activity was expressed as nmoles of α-ketobutyrate produced during 1 min incubation at 37 °C per 1 mg of protein.

The activity of MPST was assayed according to the method of Valentine and Frankelfeld [24], following a procedure described by Wróbel and others [25]. The incubation mixture contained 250 µL 0.12 M sodium phosphate buffer (pH 8.0), 50 µL 0.5 M sodium sulfite, 50 µL 0.15 M dithiothreitol, 50 µL homogenate, 50 µL H_2_O, and 50 µL 0.1 M 3-mercaptopyruvate acid sodium salt, in a final volume of 500 µL. The mixture was incubated for 15 min. To stop the reaction, 250 µL of 1.2 M PCA were added. The samples were centrifuged at 1600× *g* for 5 min, and 100 µL of supernatant was transferred to 1350 µL mixture containing 1200 µL 0.12 M sodium phosphate buffer (pH 8.0), 100 µL 0.1 M *N*-ethylmaleimide, and 50 µL NADH (5 mg/mL). After equilibration at 37 °C, 2.5 µL of l-lactic dehydrogenase (7 IU) was added, and the decrease in absorbance at 340 nm was measured. The enzyme activity was expressed as nmoles of pyruvate produced during 1 min incubation at 37 °C per 1 mg of protein.

The TST activity was assayed by the Sörbo’s method [26], with modification described by Wróbel and others [25]. The incubation mixtures contained 200 µL 0.125 M sodium thiosulfate, 100 µL 0.2 M potassium phosphate, 100 µL homogenate, and 100 µL 0.25 M potassium cyanide, in a final volume of 500 µL. After 5 min incubation at room temperature 100 µL of 38% formaldehyde, and 500 µL 0.25 M ferric nitrate reagent were added. The control samples were prepared in the same way as the examined samples, but formaldehyde was added before potassium cyanide. Thiocyanate (SCN^−^) was estimated colorimetrically at 460 nm. The enzyme activity was expressed as nmoles of SCN^−^, which formed during 1 min incubation at room temperature per 1 mg of protein.

The activity of CBS was examined in homogenates after 30 min of the incubation at 37 °C in the presence of d,l-homoserine (HSer) as a substrate using RP-HPLC method described in previous paper [27]. The CBS activity was expressed as pmoles of cystathionine formed during 1 min incubation at 37 °C per 1 mg of protein.

### 2.7. Sulfane Sulfur

Sulfane sulfur was determined by the method of Wood [28], based on cold cyanolysis and colorimetric detection of the ferric thiocyanate complex ion. Incubation mixtures in a final volume of 880 µL contained 20 µL 1 M ammonia solution, 20 µL homogenate, 740 µL H_2_O, and 100 µL 0.5 M potassium cyanide. The control samples were prepared in the same way as the examined samples, but the homogenate was replaced with water. After 45 min incubation at room temperature 20 µL 38% formaldehyde, and 40 µL 0.25 M ferric nitrate reagent were added, after which thiocyanate was estimated calorimetrically at 460 nm. The sulfane sulfur level was expressed as nmoles of SCN^−^ produced per 1 mg of protein.

### 2.8. Protein Level

The total protein content was determined by the method of Lowry and others [29], which is based on the reaction of peptide bonds and aromatic amino acids included in proteins with Folin–Ciocalteu reagent in alkaline environment in the presence of copper ions. Crystalline bovine serum albumin was used as a standard.

### 2.9. Low Molecular Sulfur-Containing Compounds Determination Using RP-HPLC

The level of low molecular sulfur-containing compounds, such as reduced (GSH) and oxidized (GSSG) glutathione, cysteine (CSH), cystine (CSSC), and cystathionine were determined by RP-HPLC method of Dominick and others [30] with the modifications described by Bronowicka-Adamska and others [31]. Standard curves were generated in the supernatant obtained from tissue homogenates in the range from 13 to 75 nmoles of each compound per mL.

### 2.10. Detection of H_2_S Production in Tissue Homogenates

H_2_S production was determined essentially as described in previous studies [32,33]. Tissue samples were homogenized in 100 mM ice-cold potassium phosphate buffer (pH 7.4) (1 g tissue/5 mL solution). The reaction mixture in total volume 500 μL contained 20 μL 10 mM l-cysteine, 20 μL 2 mM pyridoxal 5-phosphate, 30 μL saline, and 430 μL tissue homogenates. The reaction was initiated by transferring the microtubes with prepared mixtures from ice to a water bath at 37 °C. In some samples, the enzymatic reaction was immediately stopped by the addition of 250 μL 10% trichloroacetic acid in order to denature proteins. After 30 min incubation, 250 μL 1% zinc acetate was added to trap the evolved H_2_S, followed by 10% trichloroacetic acid. Then, 133 μL of 20 mM *N*,*N*-dimethyl-p-phenylenediamine sulfate in 7.2 M HCl and 133 μL of 30 mM FeCl_3_ in 1.2 M HCl were added. The absorbance of the resulting solution was measured at 670 nm after 15 min incubation in room temperature using a 96-well microplate reader. The basal concentration of H_2_S was determined using incubation mixture with trichloroacetic acid added at time zero prior to the addition of cysteine. All samples were assayed in duplicate. The H_2_S concentration of each sample was calculated by a standard curve of NaHS at the concentration range from 3.25 to 250 μM. Results were expressed as nmoles of H_2_S produced during 1 min incubation at 37 °C per 1 mg of protein.

### 2.11. Statistical Analysis

All results were represented as arithmetic means with standard deviations (S.D.). Significance of the differences between experimental groups were determined using the Mann–Whitney test (*p* < 0.05). All experiments were repeated at least three times.

## 3. Results

The experiments were conducted on the livers of rats divided into four research groups—the tissues were collected from rats with normal blood pressure (WKY) and rats with arterial hypertension (SHRs) divided additionally by age (16- or 60-week-old).

In the groups of 16-week-old rats there was no difference in body weight between normotensive and hypertensive rats. However, the WKY rats showed higher food intake, lower water intake, and lower urine output. In turn, no differences in body weight, food intake, water intake, and urine output were observed in the groups of 60-week-old rats (detailed parameters in the study by Huc and others) [18]. Mean arterial blood pressure in anaesthetized SHRs was 120.98 ± 5.03 and 101.6 ± 4.34 mmHg in 60- and 16-week-old animals, respectively. In anaesthetized WKY mean arterial blood pressure was 81.79 ± 2.19 and 83.73 ± 4.46 mmHg in 60- and 16-week-old rats, respectively [18].

**Figure 2:**
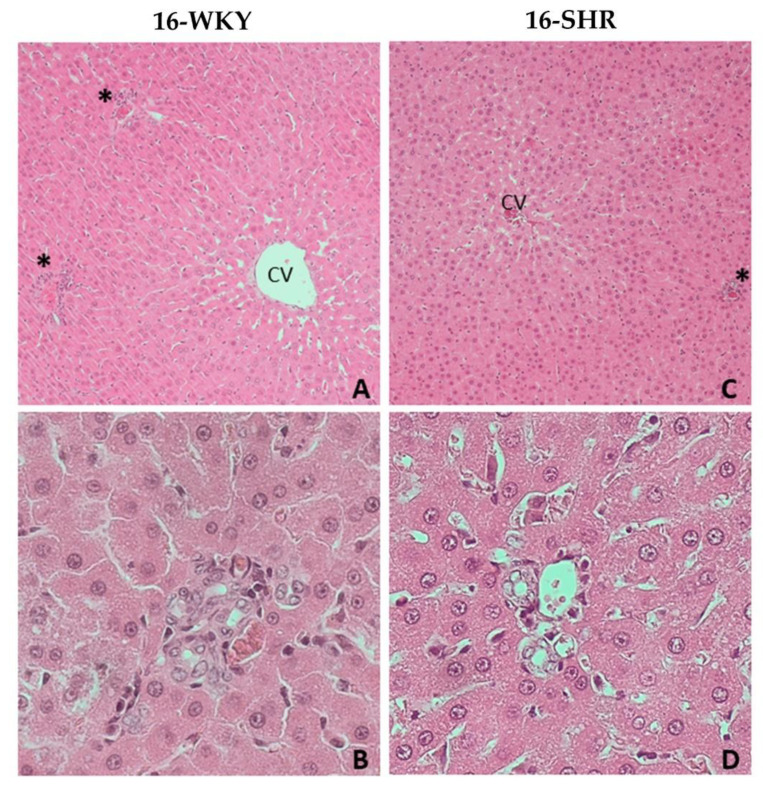
Histopathological picture of livers of 16-week-old rats. Picture shows the results of histopathological examination of the following groups: 16-week-old Wistar Kyoto rats (16-WKY) and 16-week-old Spontaneously Hypertensive Rats (16-SHR). (**A**,**C**)—liver parenchyma at magnification of the lens 10×; (**B**,**D**)—portal fields at magnification of the lens 40×. CV—central vein; *—portal field.

**Figure 3:**
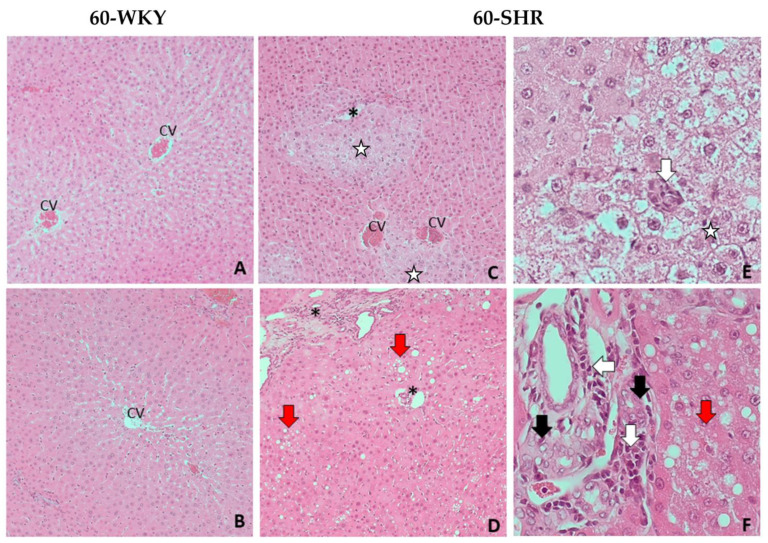
Histopathological picture of livers of 60-week-old rats. Picture shows the results of histopathological examination of the following groups: 60-week-old Wistar Kyoto rats (60-WKY) and 60-week-old Spontaneously Hypertensive Rats (60-SHR). (**A**–**D**)—liver parenchyma at magnification of the lens 10×; (**E**)—hepatocytes with hydrophic degeneration at magnification of the lens 40×; (**F**)—portal fields at magnification of the lens 40×. CV—central vein; *—portal field; white arrow—foci of mononuclear cell infiltration; black arrow—cholangiocyte hyperplasia; red arrow—steatosis foci; white star—foci of hydrophic degeneration.

There were no significant pathomorphological changes in the livers of 16-week-old rats, both WKY and SHR group (Figure 2). Histopathological examination of livers from 60-WKY group showed only minimal, focal hydropic degeneration of hepatocytes (Figure 3A,B). In 60-SHR group moderate pathological changes in hepatocytes, including intra-parenchymal hydrophic degeneration of lobular centers, edema and enlargement of hepatocytes, cariomegaly and binucleus were found (Figure 3C,E). Moreover, hepatocytes around the portal fields (zone 1 of the lobules) revealed features of mild, mixed steatosis, both macrovesicular (large-droplet) and microvesicular (small-droplet) (Figure 3D,F). Bile duct hyperplasia and mononuclear cell infiltration were found within the portal fields (Figure 3F). In the liver parenchyma of 60-week-old SHRs diffuse small foci of mononuclear cell infiltration were observed (Figure 3E).

**Figure 4:**
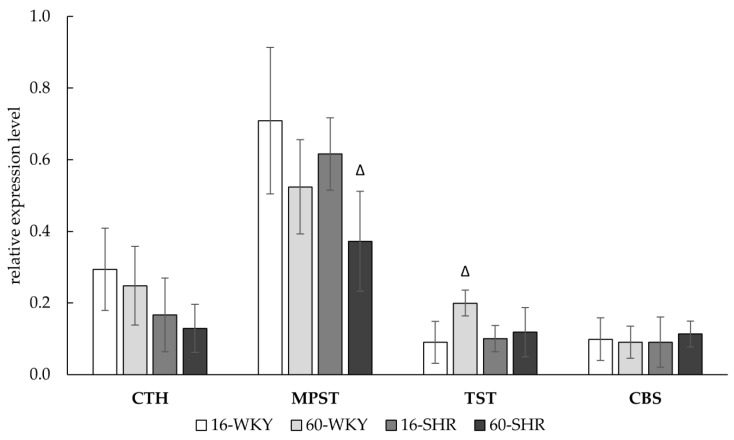
Relative expression level of CTH, MPST, TST, and CBS gene in rats’ livers. Rats were divided into the following groups: 16-week-old Wistar Kyoto rats (16-WKY), 60-week-old Wistar Kyoto rats (60-WKY), 16-week-old Spontaneously Hypertensive Rats (16-SHR), and 60-week-old Spontaneously Hypertensive Rats (60-SHR). Bands were normalized using GAPDH gene, the mean of which was taken as equal to one. Values represent an arithmetic mean ± S.D. of three animals. ^Δ^ *p* < 0.05 16-week-old vs. 60-week-old (Mann–Whitney test).

Results of experiments confirmed the expression (mRNA levels) of four enzymes—CTH, MPST, TST, CBS—involved in H_2_S metabolism, in liver (Figure 4). In the studied groups, no significant changes in the expression of CTH gene were found, but there was a tendency to decrease the CTH expression with hypertension and age. For the MPST gene expression, a decrease in 60-week-old rats was observed, with a statistically significant decrease in 60-SHR group compared to 16-SHR group. Expression of TST gene was similar in all of the investigated groups except 60-WKY group where the expression level was significantly higher than in the 16-WKY group. The relatively low level of CBS gene expression was similar in all of the study groups.

Table 1 shows the activity of sulfur enzymes and sulfane sulfur levels. The CTH activity, expressed as nmoles of product formed during 1 min per 1 mg of protein, was significantly higher in 60-week-old hypertensive rats compared with WKY rats of the same age and with 16-week-old SHRs. In 60-WKY group the activity of MPST and TST was significantly higher than in the 16-WKY group, what in case of the TST activity was correlated with the higher gene expression. In the livers of SHRs a statistically significant decrease in MPST activity was found in 60-week-old rats compared to 16-week-old animals (in accordance with relative gene expression level). In addition, it was observed that the activity of TST in the groups of 16-week-old rats is significantly higher in animals with hypertension. CBS activity in all four groups was low or undetectable, and in accordance with low mRNA expression levels. Sulfane sulfur level significantly increased in 60-week-old rats regardless of absence or presence of arterial hypertension. However higher increase was observed in SHRs group where the level of sulfane sulfur in 60-week-old rats was more than 1.5 times higher than in 16-week-old rats. Additionally, sulfane sulfur level in 16-week-old SHRs was significantly lower than in WKY rats of the same age.

**Figure 5:**
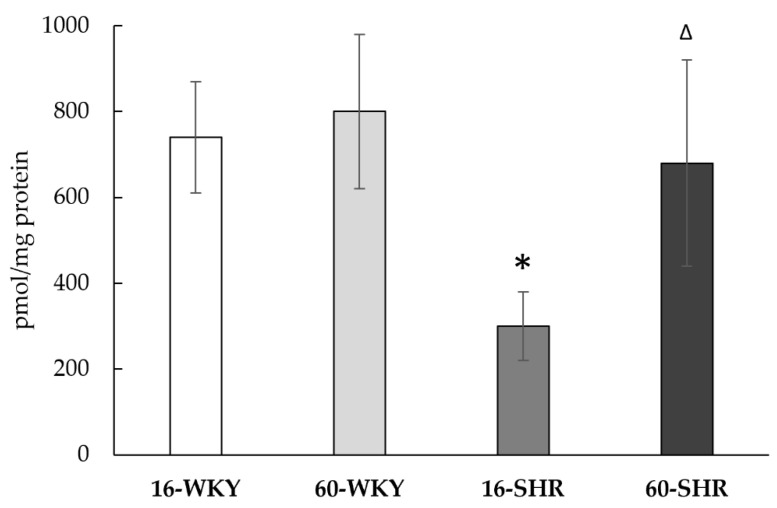
The level of cystathionine in rats’ livers. Rats were divided into the following groups: 16-week-old Wistar Kyoto rats (16-WKY), 60-week-old Wistar Kyoto rats (60-WKY), 16-week-old Spontaneously Hypertensive Rats (16-SHR), and 60-week-old Spontaneously Hypertensive Rats (60-SHR). Values represent an arithmetic mean ± S.D. of 3–4 animals. Each value is the mean of 8–12 repeats. * *p* < 0.05 WKY vs. SHR in the same age, ^Δ^ *p* < 0.05 16-week-old vs. 60-week-old (Mann–Whitney test).

The level of low molecular weight sulfur compounds in the experimental groups is shown in Table 2 and Figure 5. The results show that both hypertension and age may affect the oxidative-reduction state of the liver. In hypertensive rats the reduced glutathione (GSH) level was two times lower in the 60-SHR group than in the 16-SHR group. Inversely in the 60-WKY group level of GSH was significantly higher (over 2.5 times) than in the 16-WKY group. Furthermore, in groups of 16-week-old rats the concentration of GSH was significantly higher in rats with hypertension. The level of oxidized glutathione (GSSG) was significantly higher in WKY rats than SHRs and did not change depending on age. However, in the groups of rats with arterial hypertension a significantly higher GSSG concentration in 60-week-old compared to 16-week-old rats was noticed. Significantly higher levels of cysteine (CSH) in the liver have been associated with the occurrence of hypertension in rats; the highest CSH level was marked in the 60-SHR group. The concentration of cystine (CSSC) was similar in all experimental groups except the 60-SHR group where its level was significantly higher than in the 16-SHR and 60-WKY groups. Level of cystathionine was significantly lower in 16-week-old SHRs what was correlated with undetectable value of CBS activity (Table 1), which catalyzes its formation (Figure 1).

**Figure 6:**
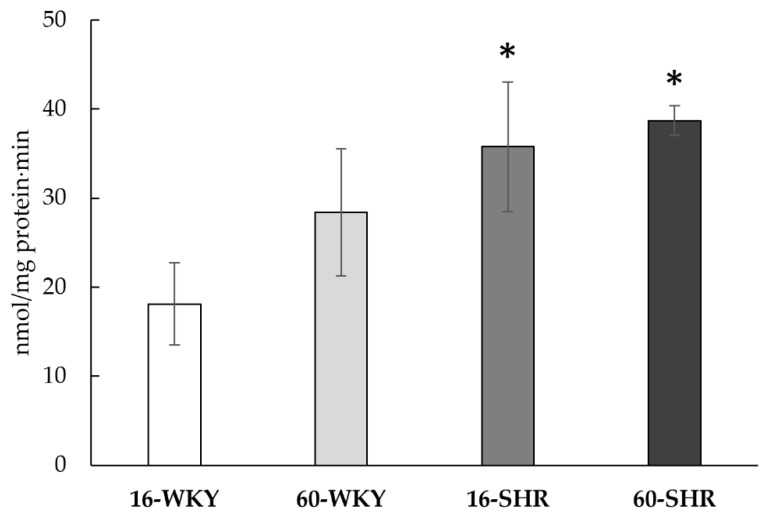
H_2_S production in rats’ livers. Rats were divided into the following groups: 16-week-old Wistar Kyoto rats (16-WKY), 60-week-old Wistar Kyoto rats (60-WKY), 16-week-old Spontaneously Hypertensive Rats (16-SHR), and 60-week-old Spontaneously Hypertensive Rats (60-SHR). Values represent an arithmetic mean ± S.D. of three animals. Each value is the mean of 8–12 repeats. * *p* < 0.05 WKY vs. SHR in the same age (Mann–Whitney test).

The liver’s ability to generate hydrogen sulfide was confirmed in all the studied groups (Figure 6). Tissue ability to produce H_2_S was significantly higher in both SHRs groups than in both WKY groups (respectively by 98% in 16-week-old and by 36% in 60-week-old rats).

## 4. Discussion

The effect of hydrogen sulfide on blood pressure relies on the relaxation of blood vessels by hyperpolarization of smooth muscle cells associated with the opening of ATP-sensitive potassium channels (K_ATP_) [4]. A study by Zhao and others [34] has shown that peripheral administration of hydrogen sulfide reduces blood pressure in rats. In turn, the development of hypertension was observed in mice with knockout CTH gene [35]. In the case of liver, Tan and others [36] have shown that supplementation of NaHS, a donor of H_2_S, protects liver function, reduces hepatic fibrosis and portal hypertension in rats treated with carbon tetrachloride.

**Figure 7:**
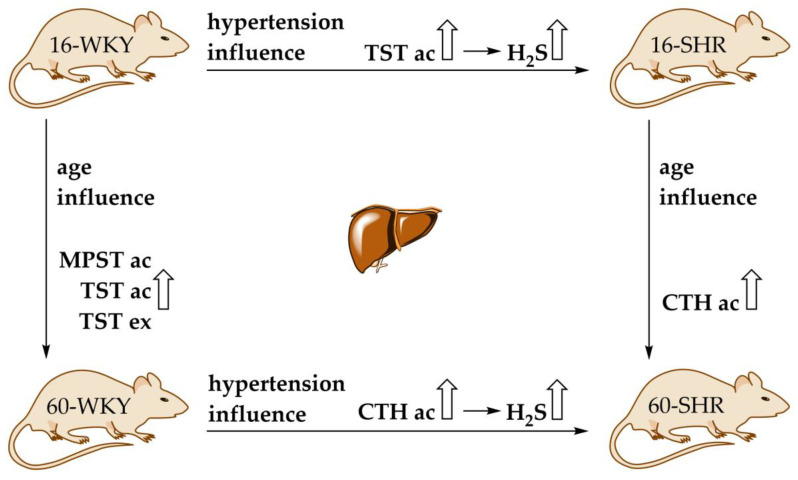
Hypertension and age influence for activity and gene expression of sulfur enzymes, and H_2_S generation in rats’ livers. 16-WKY: 16-week-old Wistar Kyoto rats; 60-WKY: 60-week-old Wistar Kyoto rats; 16-SHR: 16-week-old Spontaneously Hypertensive Rats; 60-SHR: 60-week-old Spontaneously Hypertensive Rats; CTH: cystathionine gamma-lyase; MPST: 3-mercaptopyruvate sulfurtransferase; TST: rhodanese; ac: activity; ex: gene expression.

There are many reports dealing with the two enzymes—CTH and CBS—that affect the production of hydrogen sulfide in the liver [13,37,38]. In this study we showed the gene expression and activity of four enzymes involved in hydrogen sulfide production (Figure 4, Table 1) and confirmed H_2_S generation (Figure 6) in rat liver. Our research showed that arterial hypertension and aging affect the hepatic sulfur metabolism and H_2_S production in rats (Figure 7). Moreover, the obtained results suggest that MPST and TST (beside CTH) may have a significant impact on the sulfur-containing compounds formation in the rat liver, and above all on the regulation of the redox state of the tissue.

Histopathological examination showed hydropic degeneration in the livers of 60-week-old rats, in both WKY and SHR groups (Figure 3). It results from disturbances in the integrity and function of the cell membrane, especially in ion channels, which leads to accumulation of intracytoplasmic fluid in the form of vacuoles and cell swelling. It could be caused by the direct damage to the membrane, e.g., by ROS or disturbance of mitochondrial metabolism and a decrease in the availability of ATP, necessary for the proper function of the membrane channels [39]. The second degenerative change in hepatocytes was steatosis, which results from the accumulation of lipids in the cytoplasm in the form of vacuoles. Typically, hepatocyte fatty disorders are associated with a dysfunction of the mitochondrial lipid metabolism [40,41]. Simultaneously accumulating lipids can be a source of ROS, which leads to further cell damage and necrosis [42]. Importantly, the described changes are located in zone 1 of the hepatic lobule (periportal localization), which is characterized by intense oxidative processes such as gluconeogenesis, beta-oxidation of fatty acids or cholesterol synthesis [43]. Caromegaly/caryocytomegaly and biliary duct hyperplasia are typical senile changes in hepatocytes, and their severity may increase with the presence of other pathological processes involving liver cells. Moreover, the infiltration of mononuclear cells and cholangiocyte hyperplasia are most likely secondary to the processes described in hepatocytes and may indicate the occurrence of single cell necrosis of hepatocytes [44].

In this experiment, no significant effect of hypertension on the gene expression of sulfur enzymes in both age groups was observed (Figure 4). However, hypertension affected sulfur enzymes activity and the levels of sulfur compounds (Table 1 and Table 2). In 16-week-old SHRs group high TST activity (Table 1) was correlated with high GSH level (Table 2) and both values were significantly higher compared to WKY rats of the same age. Additionally, GSSG level in the 16-SHR group, decreased more than three times compared to the 16-WKY group. These results are in line with Nakajima’s suggestions [45], that prolonged stress may increase both rhodanese and MPST activity, which in turn can affect GSH level and give an antioxidant effect. CBS activity, noticed in the 16-WKY group, was not detected in the 16-SHR group (Table 1) and was correlated with low level of cystathionine (Figure 5), which suggests that hypertension causes a decrease in the activity of this enzyme in 16-week-old rats. On the other hand, in the case of the 60-SHR group, we noticed a decrease of MPST activity (Table 1) and GSH level (Table 2) compared to 60-WKY rats group. However, in this group CTH activity increased significantly in relation to WKY rats of the same age and 16-week-old SHRs (Table 1), which suggests that in the 60-SHR group CTH may be the main enzyme responsible for the production of H_2_S.

Both in the group of SHRs and WKY rats, a significant influence of age on the activity of sulfur enzymes was noticed. In the 60-SHR group a decrease in the MPST gene expression (Figure 4) and activity (Table 1) compared to the 16-SHR group, correlated with a lower level of reduced glutathione and higher level of GSSG (Table 2). In turn, in normotensive rats a statistically significant increase in MPST and TST activity (Table 1), and also increase in TST gene expression (Figure 4) in 60-week-old rats in comparison to 16-week-old animals was observed. Simultaneously the level of GSH increased more than 2.5 times (Table 2), which is consistent with Nakajima’s suggestions [45], that high rhodanese and MPST activity increases GSH content. Additionally, higher concentration of reduced glutathione has been observed in the livers of 23-month-old Wistar rats compared to 7-week-old and 8-month-old rats by Yang and others [46]. These results indicate that the high activity of mitochondrial sulfurtransferases is a mechanism compensating oxidative stress related with age but only in normotensive rats.

Hypertension influenced the levels of sulfane sulfur—one of the precursors of hydrogen sulfide (Figure 1)—in rats from both age groups compared to WKY rats (Table 1). The content of sulfane sulfur in 16-week-old rat groups was significantly lower in SHRs than in WKY rats, opposite than in the groups of 60-week-old rats. Both, in normotensive and in hypertensive rats, the levels of sulfane sulfur change with age and in the 60-WKY group correlated with increased rhodanese and MPST activity (Table 1). Accumulation of sulfane sulfur in livers of 60-week-old rats may be related to its high fat solubility [47], the amount of which increases with age in the liver [9,17]. This effect was enhanced in the livers of rats with hypertension, in the case of which the histopathological examination confirms the presence of steatosis foci (Figure 3D,F).

In turn, the level of the main substrate for H_2_S production—cysteine (Figure 1)—was significantly higher in the SHRs in both age groups (Table 2) compared to WKY rats. Moreover, the level of total cysteine (calculated as a sum of 2 CSSC content + CSH content) was significantly higher in the 60-SHR group, which was influenced by a significantly high level of CSSC. Lu [48] reported that under oxidative stress hepatocytes can capture extracellular cysteine, which is then oxidized to cystine. Furthermore, cysteine is an essential amino acid for glutathione biosynthesis [49], so in the 60-SHR group low level of total glutathione (2GSSG+GSH) (Table 2) explains high concentration of free cysteine.

Arterial hypertension is associated with dysfunction of endothelial cells, disturbance of NO balance, and increased production of ROS [50]. Similarly, advanced age promotes the accumulation of ROS in liver cells, leading to impairment of the respiratory chain complexes [9], which results in a disturbance of the oxygen balance, and consequently a disturbance of the body’s energy metabolism. Additionally, aging of rat liver contributes to a reduction in oxygen uptake by that tissue [51]. In turn, studies using human embryonic kidney cells HEK 293 showed that low oxygen availability promotes the production of hydrogen sulfide in mitochondria [52]. In this study we observed that the livers of SHRs of both age groups can generate significantly more hydrogen sulfide than WKY rats’ livers (Figure 6). Vasorelaxation dependent on H_2_S is important in improving the hepatic microcirculation [14], so the increased capacity to produce hydrogen sulfide in the SHRs livers may be a mechanism to compensate for the potential microvessel dysfunction caused by hypertension. Furthermore, a high hydrogen sulfide generation capacity in the 60-WKY group was observed, although this was not a statistically significant change compared to the 16-WKY group. These results are in line with previous theories that endogenous hydrogen sulfide in mammalian cells under hypoxic or stressed conditions can be an energy source for adenosine triphosphate (ATP) production [53,54,55]. In tissues exposed to chronic oxidative stress high concentration of H_2_S may compensate energy deficiencies.

## 5. Conclusions

The study showed the gene expression and activity of four enzymes involved in the metabolism of sulfane sulfur and hydrogen sulfide: CTH, MPST, TST, and CBS in rat livers. The influence of hypertension on changes in the metabolism of sulfur compounds in younger rats is large and is manifested, compared to the corresponding WKY group, by significantly higher TST activity, lower sulfane sulfur level and greater possibilities for H_2_S formation, but also undetectable level of CBS and significantly lower level of cystathionine. Inversely, in the livers of older SHRs, CBS activity and high CTH activity was demonstrated. High levels of MPST and TST were noticed, in turn, in older WKY rats.

The obtained results confirm that hypertension and aging affect the metabolism of sulfur-containing compounds in the rats’ livers in different ways. They indicate a significant role of mitochondrial sulfurtransferase TST in hypertensive younger rats, and CTH in older hypertensive rats, and also MPST and TST in compensating for changes caused by aging of normotensive rats (Figure 7).

## Figures and Tables

**Table 1:** The activity of MPST, CTH, TST, and CBS, and the sulfane sulfur level in rats’ livers.

Experimental Group	CTH	MPST	TST	CBS	Sulfane Sulfur
	nmol/mg protein·min			pmol/mg protein·min	nmol/mg protein
16-WKY	14.7 ± 2.8	6713 ± 551	5244 ± 775	1.8 ± 0.8	249 ± 46
60-WKY	16.9 ± 3.3	7827 ± 517 ^Δ^	8448 ± 539 ^Δ^	2.7 ± 0.7	288 ± 23 ^Δ^
16-SHR	16.7 ± 2.7	6684 ± 530	9545 ± 1383 *	ND	223 ± 19 *
60-SHR	21.4 ± 2.9 *^Δ^	6164 ± 592 *^Δ^	8120 ± 2822	2.3 ± 0.4	358 ± 87 *^Δ^

Rats were divided into the following groups: 16-week-old Wistar Kyoto rats (16-WKY), 60-week-old Wistar Kyoto rats (60-WKY), 16-week-old Spontaneously Hypertensive Rats (16-SHR), and 60-week-old Spontaneously Hypertensive Rats (60-SHR). Values represent an arithmetic mean ± S.D. of 3–4 animals. Each value is the mean of 15–24 repeats. * *p* < 0.05 WKY vs. SHR in the same age, ^Δ^ *p* < 0.05 16-week-old vs. 60-week-old (Mann–Whitney test). ND: not detected.

**Table 2:** The level of reduced and oxidized glutathione, and the level of cysteine and cystine in rats’ livers.

Experimental Group	GSH	GSSG	Total Glutathione (2GSSG+GSH)	CSH	CSSC	Total Cysteine (2CSSC+CSH)
	nmol/mg protein					
16-WKY	7.6 ±1.8	11.1 ± 3.0	28.4 ± 5.5	0.4 ± 0.3	4.3 ± 0.7	9.0 ± 1.4
60-WKY	20.2 ± 3.6 ^Δ^	10.3 ± 2.4	41.4 ± 6.8 ^Δ^	1.0 ± 0.6	4.1 ± 1.0	9.2 ± 2.8
16-SHR	32.3 ± 5.7 *	2.9 ± 0.7 *	38.3 ± 7.2 *	3.0 ± 0.7 *	3.9 ± 1.0	10.8 ± 2.5
60-SHR	15.6 ± 2.9 *^Δ^	4.2 ± 0.9 *^Δ^	21.9 ± 3.5 *^Δ^	7.9 ± 2.8 *^Δ^	7.3 ± 1.6 *^Δ^	22.2 ± 6.0 *^Δ^

Rats were divided into the following groups: 16-week-old Wistar Kyoto rats (16-WKY), 60-week-old Wistar Kyoto rats (60-WKY), 16-week-old Spontaneously Hypertensive Rats (16-SHR), and 60-week-old Spontaneously Hypertensive Rats (60-SHR). Values represent an arithmetic mean ± S.D. of 3–4 animals. Each value is the mean of 8–12 repeats. CSH: cysteine; CSSC: cystine; GSH: reduced glutathione; GSSG: oxidized glutathione. * *p* < 0.05 WKY vs. SHR in the same age, ^Δ^ *p* < 0.05 16-week-old vs. 60-week-old (Mann–Whitney test).

## Data Availability

All relevant data are presented in this paper.
